# A single-step method for RNA isolation from tropical crops in the field

**DOI:** 10.1038/srep38368

**Published:** 2016-12-06

**Authors:** J.-C. Breitler, C. Campa, F. Georget, B. Bertrand, H. Etienne

**Affiliations:** 1 CIRAD, UMR IPME, 911 Avenue Agropolis, BP 64501, 34394, Montpellier, France; 2 IRD, UMR IPME, 911 Avenue Agropolis, BP 64501, 34394, Montpellier, France

## Abstract

The RNAzol RT reagent was used to provide pure RNA from human cells. We develop a protocol using RNAzol RT reagent to extract pure RNA from plants tissues and demonstrate that this RNA extraction method works not only at room temperature but also at elevated temperatures and provides the simplest and most effective single-step method to extract pure and undegraded RNA directly from tropical plants in the field. RNA extraction directly in a complex field environment opens up the way for studying gene-environment interactions at transcriptome level to decipher the complex regulatory network involved in multiple-stress responses.

In 2010, Chomczynski and co-workers published a new approach to single-step RNA extraction using RNAzol RT reagent, which does not employ chloroform-induced phase separation to obtain pure RNA^1^. With the introduction of RNAzol RT, the single-step method has been improved to provide RNA that is ready to use for reverse transcription polymerase chain reaction (RT-PCR) without additional purification or treatment with DNase. In that publication, the authors mentioned that this extraction technique works at room temperature. On this basis, we undertook to determine whether this method could also be efficient at high temperatures in order to use single-step RNA extraction directly in tropical fields.

The acclimation of plants to field conditions is a coordinated response involving hundreds of genes and a complex regulatory network. These responses are governed by interactions between different environmental factors and the developmental stage of the plant^2^. The complex field environment with its heterogenous conditions and stress combinations, in a context of global climate change, represents a new challenge in understanding plant adaptation mechanisms to define new breeding programmes^3^. Trees are exposed to myriad single and combined stresses with varying strengths and durations throughout their lifetime, and many of the simultaneous and successive stress factors strongly interact. While much progress has been made in understanding the effects of single stresses on tree performance, multiple interacting stress effects cannot be adequately assessed from a combination of single-factor analyses. In particular, global change brings about novel combinations in the severity and timing of different stresses, whose effects on tree performance are currently hard to predict. In this context, the study of stress combinations directly in the field appears essential for deciphering interactions between genes and the environment^4^.

The recent development of novel high-throughput DNA sequencing methods has provided a new powerful method for transcriptome analysis. Because RNA-Seq provides a far more precise measurement of both the transcriptional structure and level of expression for each gene than other methods, this technique is perfectly suited to deciphering complex regulatory networks involved in multiple-stress responses^5^. Indeed, pathway databases and metabolic networks reconstructed from transcriptomics sequence data can help in discovering how developmental and environmental cues affect primary and secondary metabolism^6^. Sequencing technologies have placed a wide range of transcriptomic analyses within the capabilities of many laboratories. While library construction encounters some technical difficulties, the quality of the library primarily depends on that of the extracted RNA, which is easy to obtain in a well-equipped laboratory from samples frozen in liquid nitrogen. However, the difficulty in extracting high-quality RNA from samples taken from experimental field plots in many tropical countries, where RNA sequencing facilities are not available without breaking the cold chain, greatly limits the use of these technologies and, consequently, the biological outcomes that could be achieved from a complex field environment. A better attempt needs to be made to develop field sampling for molecular analysis because, in many tropical regions, it is impossible to procure liquid nitrogen, which prevents sampling for RNA extraction. Based on the RNAzol RT single-step method for the isolation of RNA^1^, we developed an innovative approach that takes into consideration hardware requirements and extraction costs, in order to propose a new versatile and effective method for extracting high-quality plant RNAs directly in the experimental plots of tropical fields.

## Results

### Evaluation of the quality of RNA extracted in field

**Figure 1:**
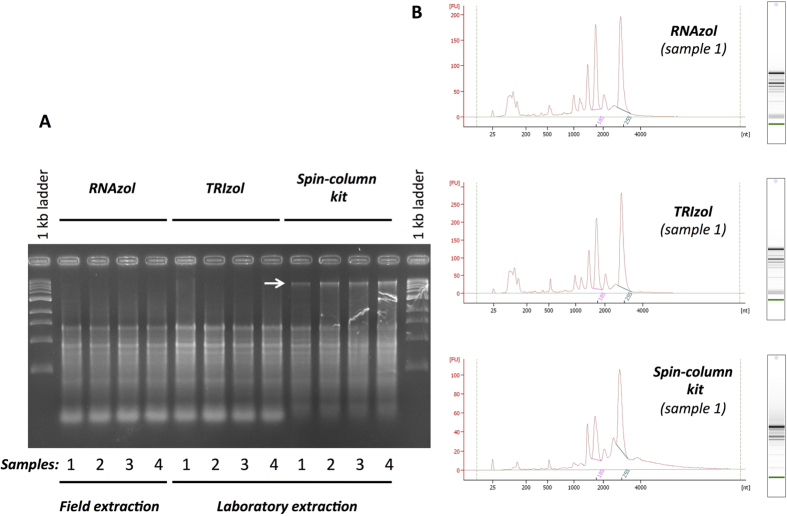
Comparison of the effectiveness of RNAzol RT (RNA extraction in field) and TRIzol Reagent or spin-columns kit (RNA extraction in laboratory with recommended cold conditions) to isolate total RNA. (**A**) Electrophoretic separation using 2% agarose gel of RNA extracted from the same four samples using the three methods. (**B**) Electropherogram and Electrophoresis of representative sample of RNA analysed with the Agilent 2100 bioanalyser system with the RNA 6000 Nano^™^ kits. The white arrow shows genomic DNA contaminants.

To evaluate the effectiveness of RNAzol RT to isolate RNA samples in field, we compared these RNA with RNA extracted from the same samples in the lab using TRIzol reagent or commercial spin-columns kit. A spectrophotometric analysis, an electrophoretic separation using agarose gel (Fig. 1A) and the Agilent 2100 bioanalyser system (Fig. 1B) show similar results (quality and quantity) when total RNA were isolated in field using RNAzol RT reagent or TRIzol reagent in the lab (without breaking the cold chain). The worst results were obtained with the spin-columns kit which is not well adapted to coffee leaves RNA extraction. The contamination with genomic DNA is strong and majority of small RNAs were lost (Fig. 1A).

### A reliable method applicable to several organs

**Figure 2:**
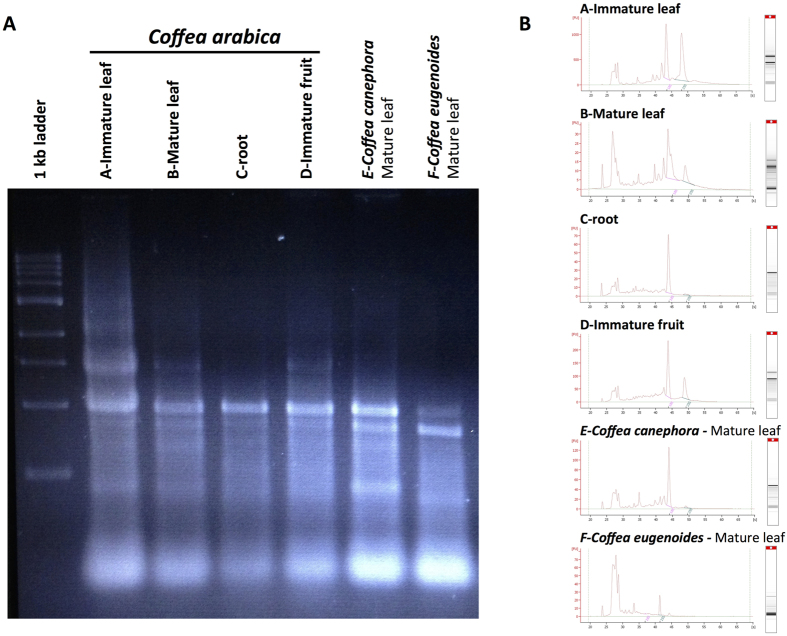
Depicts the integrity of RNA isolated at 30 °C from different *Coffea arabica* tissues (mature and immature leaf, fruit, root) and from leaves belonging to 3 different coffee species, *Coffea arabica, C. canephora* and *C. eugenoides*. The isolated RNA were analysed by electrophoretic separation using 2% agarose gel (**A**) and the Agilent 2100 bioanalyser system with the RNA 6000 Nano^™^ kits (**B**). The total RNA fraction contained ribosomal RNA, mRNA and small RNA had a RIN value ranging between 7.5 and 8.5.

The simplified protocol for single-step RNA isolation with RNAzol RT reagent was first adapted to coffee trees using mature *Coffea arabica* leaf samples to isolate pure and undegraded RNA (Fig. 2). A spectrophotometric analysis and electrophoretic separation using agarose and the Agilent 2100 bioanalyser system using more than 120 samples confirmed RNA quality with an *A*_260/280_ ratio of 1.84 ± 0.1 and a RNA integrity number (RIN) value of 8–9. The Agilent 2100 bioanalyser system confirmed the absence of genomic DNA in the RNA samples. The average quantity of total RNA extracted from 100 mg of fresh leaf tissues reached 33.4 ± 18.8 μg. Processing of RNA from more challenging plant-derived samples, such as fruits and roots, yielded RNA with an *A*_260/280_ ratio of 1.8–1.9 and a RIN value of 7–8 (using the Agilent 2100 bioanalyser system).

### Efficient use of field extracted RNAs in different transcriptomic analyses

**Figure 3:**
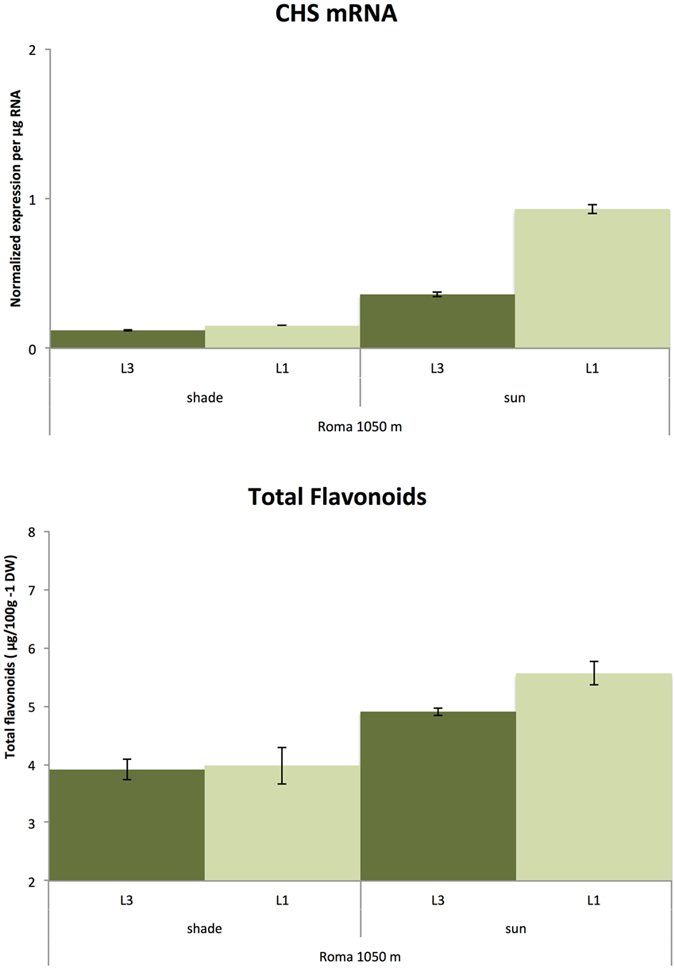
Similarity in the patterns of CHS mRNA levels measured by real-time PCR with RNAs extracted by the single-step method and flavonoid content evaluated by HPLC in leaves collected at two developmental stages (L1 and L3) from trees cultivated under full sunlight conditions (sun) versus 50% light interception (shade) in Mexico.

Quantitative RT-PCR was performed using total RNA extracted in the field from samples of *Coffea arabica* cv. Marsellesa cultivated in Mexico (Finca Roma, Coatepec, Vera-Cruz state) under full sun or shade (50% of the sun filtered). Here we present the results obtained with Chalcone synthase primers (CHS). CHS enzyme is the first committed enzyme in flavonoid biosynthesis. PCR performed with these RNA samples without reverse transcription showed no amplification of DNA in a 40-cycle PCR. In parallel, the same leaf samples were freeze-dried to be extracted with methanol/water (80/20) and analysed by HPLC for their flavonoid content. Figure 3 shows the CHS mRNA and total flavonoid content in leaf samples (first and third leaf from a plagiotropic stem apex). The level of measured CHS mRNA was consistent with the flavonoid concentration in each type of leaf. Total RNAs extracted from forty leaf samples in the field were successfully used for library construction and RNA sequencing (http://www.mgx.cnrs.fr). The samples had a quality corresponding to sequencing platform quality standards (BiSA Platform. ISO9001 certification).

### A useful method for tropical crops (coffee, cassava, rice, or maize)

**Figure 4:**
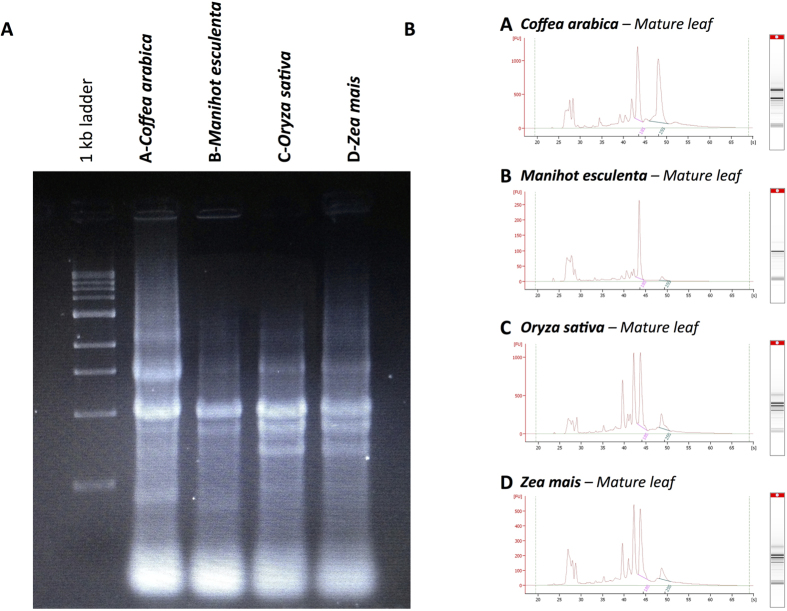
Depicts the integrity of RNA isolated at 30 °C from leaf tissue samples from *Coffea arabica, Manihot esculenta, Oryza sativa* and *Zea mais*. (**A**) The left-hand side of the figure shows the electrophoresis analysis by agarose gel. (**B**) The right-hand side of the figure shows the Agilent 2100 bioanalyser electrophoresis for each sample. The total RNA fraction had a RIN value ranging between 8 and 9.

To confirm the value of this RNA extraction method for tropical plants, other agronomically important crops were considered in this study. Mature leaf samples from cassava, rice and maize were collected in the field and total RNA was extracted following the protocol established for coffee tissues. Extracted RNAs were analysed by agarose gel electrophoresis, a spectrophotometer and a bioanalyser (Fig. 4). Similarly to coffee, both the quality and quantity of extracted RNAs were high, thus confirming the versatility of this method for tropical dicots as well as monocots.

## Discussion

The RNAzol RT single-step method has been adapted to provide pure RNA from plant tissues. This reagent isolates pure and undegraded RNA that is ready for use without DNase treatment. We have demonstrated that this RNA extraction method works not only at room temperature but also at elevated temperatures, for both monocots and dicots, and for different plant tissues. This simple and effective method can be used directly in the field for transcriptomic analysis of plants under multiple stress conditions.

## Methods

### Rapid and low-cost RNA extraction at daytime temperatures.

Basing ourselves on the work by Chomczynski and co-workers (2010), we first adapted their single-step RNA protocol for extracting RNA from coffee plant tissues, and tested its versatility and effectiveness for temperatures above 30 °C. A biological sample (80 (leaf)–250 (root) mg) is lysed in 1 ml of RNAzol RT, and the homogenate is supplemented with 400 μl of water to precipitate DNA, proteins and polysaccharides, while RNA remains soluble in the homogenate. After vortexing 10 sec and shaking for 15 min (500 rpm), the precipitated compounds are removed from the homogenate by centrifugation (15 min, 13 000 g). The supernatant is transferred to a fresh tube with 10 μl of 4-Bromoanisol and vortexed for 10 sec (this purification step is essential for plant tissues). After incubation at ambient temperature for 5 min and centrifugation for 10 min at 13 000 g, pure RNA is precipitated from the resulting supernatant by the addition of 1 volume of 2-propanol. After mixing by inversion 5 times, the solution is allowed to stand for 20 min at ambient temperature then centrifuged for 15 min at 12 500 g to pellet RNA. The pellet is washed three times with 1 ml of 75% ethanol (3 min, 6000 g) before re-suspension in 50 μl of water.

### Extraction in the field

**Figure 5:**
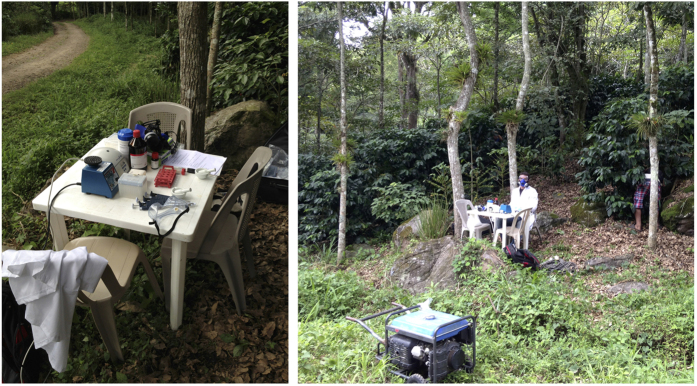
RNA extraction from coffee tree leaves in experimental plots in Nicaragua (Finca La Cumplida, Matagalpa). All equipment and devices necessary for RNA extraction can be carried in a backpack, except the generator.

The whole procedure could be achieved under the high temperatures (25–38 °C) encountered in tropical fields. Extracting total RNA from tissue samples directly in the field requires little equipment and few devices (protective equipment including breathing apparatus, generator, table and chair, pestle and mortar, pipetmans and tips, tubes, small centrifuge, vortex, sea sand, ultrapure water, 2-propanol, and 4-bromoanisol) (Fig. 5). RNA pellets were stored after the precipitation step in 75% ethanol (up to five days) and then returned to the laboratory where the final stages of extraction could be carried out with ice (the pellet was washed twice with 1 ml of 75% ethanol (centrifugation for 3 min at 6000 g), then partially dried by air-drying before re-suspension in water. Fifty samples could be successfully extracted in a working day.

### Standard RNA extraction

The total RNA extraction with TRIzol reagent is used as supplied by Invitrogen Life Technologies. QIAGEN RNeasy mini kit is used as fast commercial method to extract total RNA using spin columns (www.qiagen.com).

## Additional Information

**How to cite this article**: Breitler, J.-C. *et al*. A single-step method for RNA isolation from tropical crops in the field. *Sci. Rep.* **6**, 38368; doi: 10.1038/srep38368 (2016).

**Publisher's note:** Springer Nature remains neutral with regard to jurisdictional claims in published maps and institutional affiliations.
